# Influence of host genetics in shaping the rumen bacterial community in beef cattle

**DOI:** 10.1038/s41598-020-72011-9

**Published:** 2020-09-15

**Authors:** Waseem Abbas, Jeremy T. Howard, Henry A. Paz, Kristin E. Hales, James E. Wells, Larry A. Kuehn, Galen E. Erickson, Matthew L. Spangler, Samodha C. Fernando

**Affiliations:** 1 Department of Animal Science, University of Nebraska-Lincoln, Lincoln, NE USA; 2 Department of Animal and Dairy Science, Mississippi State University, Starkville, MS USA; 3 Meat Animal Research Center, Clay Center, NE 68933 USA

**Keywords:** Genetics, Microbiology

## Abstract

In light of recent host-microbial association studies, a consensus is evolving that species composition of the gastrointestinal microbiota is a polygenic trait governed by interactions between host genetic factors and the environment. Here, we investigated the effect of host genetic factors in shaping the bacterial species composition in the rumen by performing a genome-wide association study. Using a common set of 61,974 single-nucleotide polymorphisms found in cattle genomes (n = 586) and corresponding rumen bacterial community composition, we identified operational taxonomic units (OTUs), Families and Phyla with high heritability. The top associations (1-Mb windows) were located on 7 chromosomes. These regions were associated with the rumen microbiota in multiple ways; some (chromosome 19; position 3.0–4.0 Mb) are associated with closely related taxa (*Prevotellaceae, Paraprevotellaceae*, and *RF16*), some (chromosome 27; position 3.0–4.0 Mb) are associated with distantly related taxa (*Prevotellaceae, Fibrobacteraceae*, *RF16, RFP12, S24-7, Lentisphaerae,* and *Tenericutes*) and others (chromosome 23; position 0.0–1.0) associated with both related and unrelated taxa. The annotated genes associated with identified genomic regions suggest the associations observed are directed toward selective absorption of volatile fatty acids from the rumen to increase energy availability to the host. This study demonstrates that host genetics affects rumen bacterial community composition.

## Introduction

Complex and diverse microbial communities facilitate the degradation of nutrients within ruminants^1^. The composition of this complex microbial community is shaped by the highly dynamic physical, chemical, and predatory conditions within the rumen, and potentially by genetic factors of the host^1–6^. In turn, the microbial community contributes to the environmental conditions within the rumen and the nutrient availability to the host^2^. This complex microbial community can covert otherwise unusable organic matter into useable protein and energy and can provide up to 70% of the animal’s protein and energy needs^7^ in the form of microbial cell protein (MCP) or volatile fatty acids (VFAs) for host metabolism. Therefore, ruminal microbial diversity and abundance critically influences nutrient cycling and when inorganic nutrients and carbon are made available for host consumption. As such, differences in the microbial community can change the energy profiles available to the ruminant host and its subsequent performance.

In ruminants, the microbial population is established by successive waves where convergence of microbial populations are seen reaching a more stable population structure^8–11^. The establishment of a gut microbial community is influenced by multiple factors, including diet, environment, and host genotype^2–6,12,13^. Among these contributors, the influence of diet on gut microbial population structure is well established^14–17^. Rumen bacterial species’ and functional composition of the rumen microbiota is an important factor that contributes towards animal performance and efficiency^18^. Additionally, studies in small ruminants have demonstrated the host mucosal innate immune function to affect rumen microbial community composition^19^. With studies demonstrating host genetics to influence host immunity^20^, it is tempting to speculate in ruminants, host genetics may directly or indirectly affect rumen microbial community composition. However, our understanding of how a stable rumen microbial community assembles and what factors affect rumen microbial community composition and function is limited. One such process that is still largely unknown is how the ruminant host genotype effect rumen microbial community assembly. Recent studies have demonstrated that the rumen microbiota is influenced by host genetic factors^5,6,21^ In mammals, Benson et al. demonstrated genome-wide linkages of bacterial taxa abundance in the murine gut using Quantitative Trait Locus (QTL) mapping. While breeds did not have broad representation, a recent study suggested that microbial phylotypes can be influenced by the sire breed and can impact host metabolism^22^.

In light of these recent host-microbial association studies, a consensus is evolving that species’ composition of the gastrointestinal microbiota is a polygenic trait governed by interactions between host genetic factors and the environment. As such, genome-wide association studies (GWAS) can be used to identify host chromosomal regions and a subset of single nucleotide polymorphisms (SNP) that influence microbiome composition and function in the rumen. Here we evaluated the effect of host genetic factors in shaping the bacterial species composition in the rumen and the role of such associations on host metabolism by performing a genome wide association study using different cohorts of beef cattle totaling 586 animals. All animals were genotyped using medium to high density single nucleotide polymorphisms (SNP) chips (770 K,150 K, or 80 K) and the underlying SNPs were used for analysis of genome wide associations using the bacterial community composition as the phenotype. The rumen microbial community of all 586 animals were phenotyped by sampling and characterizing the V4 region of the 16S rRNA gene and was used as a collection of individual traits to perform GWAS to identify host chromosomal regions and subset of SNP that influence bacterial community composition and assembly within the rumen. This study provides detailed and novel insight into how host genotype in ruminants can influence rumen microbial assembly. We also quantify the genomic heritability for bacterial taxonomic traits thus identifying the degree to which they are controlled by host genetic background. This study also demonstrates the potential to utilize associations between rumen microbiota and genetic markers for use in genomic selection and marker-assisted management that can be used to improve feed efficiency, animal health and microbiome manipulation mediated by selecting for favorable microbial taxa within the rumen.

## Results

### The bacterial populations within the cattle rumen

**Figure 1:**
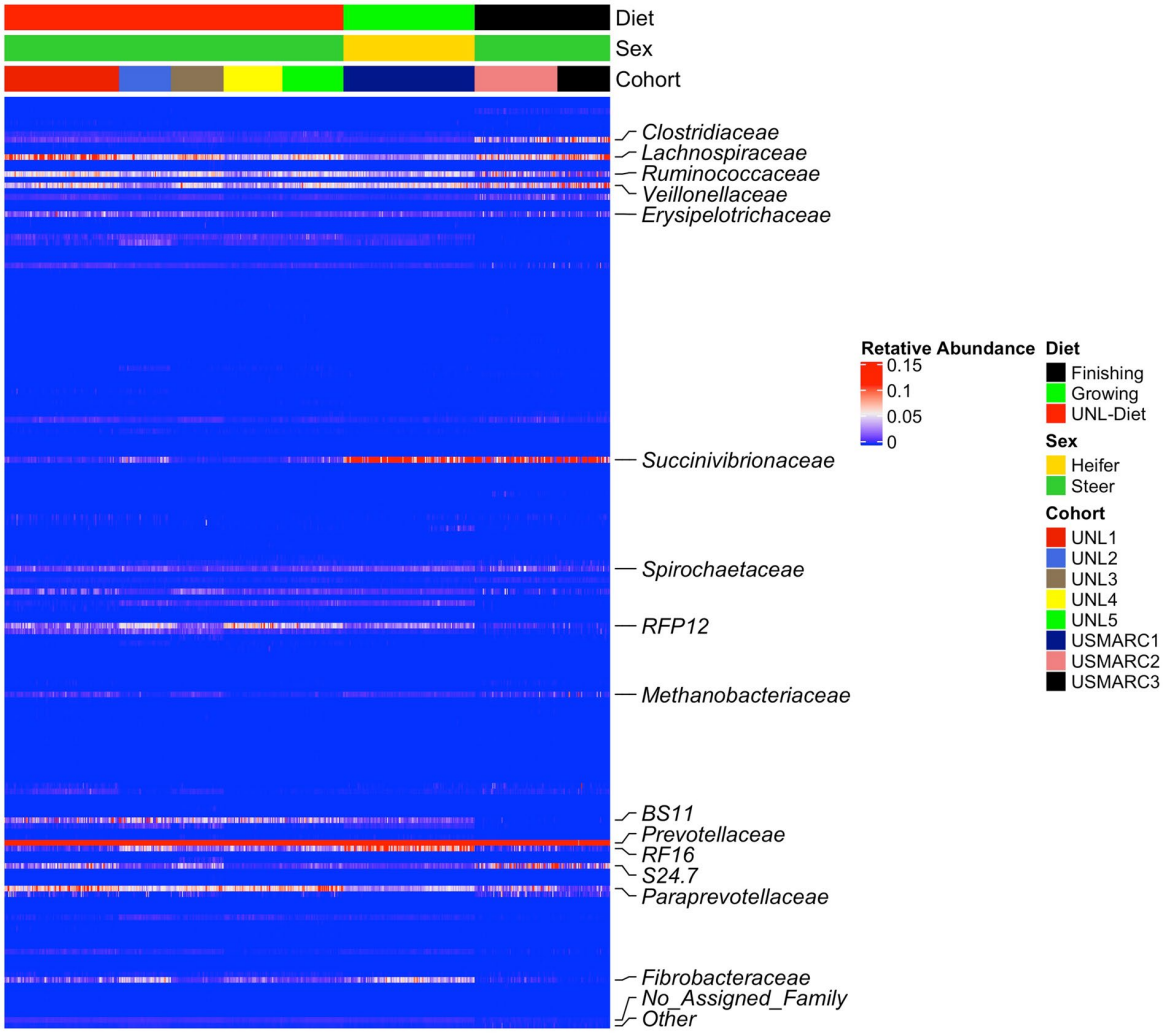
Distribution of taxa across all cohorts. The heatmap above displays the abundance and distribution of all taxa identified in at least 1% of the total animals (n = 586). The relative abundances of 237 families present within the CMM is shown above. The columns represent the samples and rows represents the relative abundance of each family. Top 17 families which accounts for nearly 90% abundance of all the families are labeled in the heatmap.

The bacterial community within the rumen microbiome of 8 cohorts were phenotyped by sequencing the V4 region of the 16S rDNA gene. The sequencing resulted in 18,992,394 quality filtered reads which included 9,755,502 reads from the USMARC cattle cohorts and 9,236,892 reads from the UNL cohorts. The rarefaction curves and goods coverage tests displayed adequate sampling depth to provide a detailed and quantitative estimate of the rumen bacterial community composition within each animal. The taxonomic classification of normalized OTUs at phylum, family and OTU level were performed using Naive Bayes classifier^23^ using the greengenes database^24^. This analysis detected 439 genera, 237 families, 131 orders, 68 classes and 32 phyla from 7,228 OTUs identified from the 586 animals sequenced. The distribution of taxa across all cohorts were similar with the exception of family Succinivibrionaceae which were greater in the USMARC cohorts (Fig. 1). The relative abundance of the major phyla included Bacteroidetes 48.85%; Firmicutes 24.95%; Proteobacteria 13.3%; Verrucomicrobia 3.14% and Tenericutes 2.24%.

The distribution of OTUs across the cohorts displayed wide animal-to-animal variation, therefore to better characterize taxa that are more conserved across animals and variable in abundance, a core measurable microbiome (CMM) was identified from the total dataset. To ensure robust repeatability of bacterial phenotyping only OTUs present in at least 1% of the animals and was also part of the CMM were used for subsequent analysis. The CMM contained 99.94% of the total reads generated and therefore represent a major portion of the rumen bacterial population. A principal coordinate analysis (PCoA) (supplementary figure S3) and PERMANOVA analysis were performed to identify combined effects of diet, sex, location on microbial community. As all factors (diet, sex, location) cannot be untangled from each other, the PERMANOVA analysis was performed using management type to reflect the collective effect of all above factors. PERMANOVA analysis displayed a significant effect of management type (p < 0.001) on microbial community composition. Therefore, in the model used for GWAS analysis, we included contemporary group (concatenation of management type and year) as a fixed effect to adjust for the variation in the microbial community composition resulting from management type.

### Rumen bacterial community behaves as a “polygenic trait”

**Figure 2:**
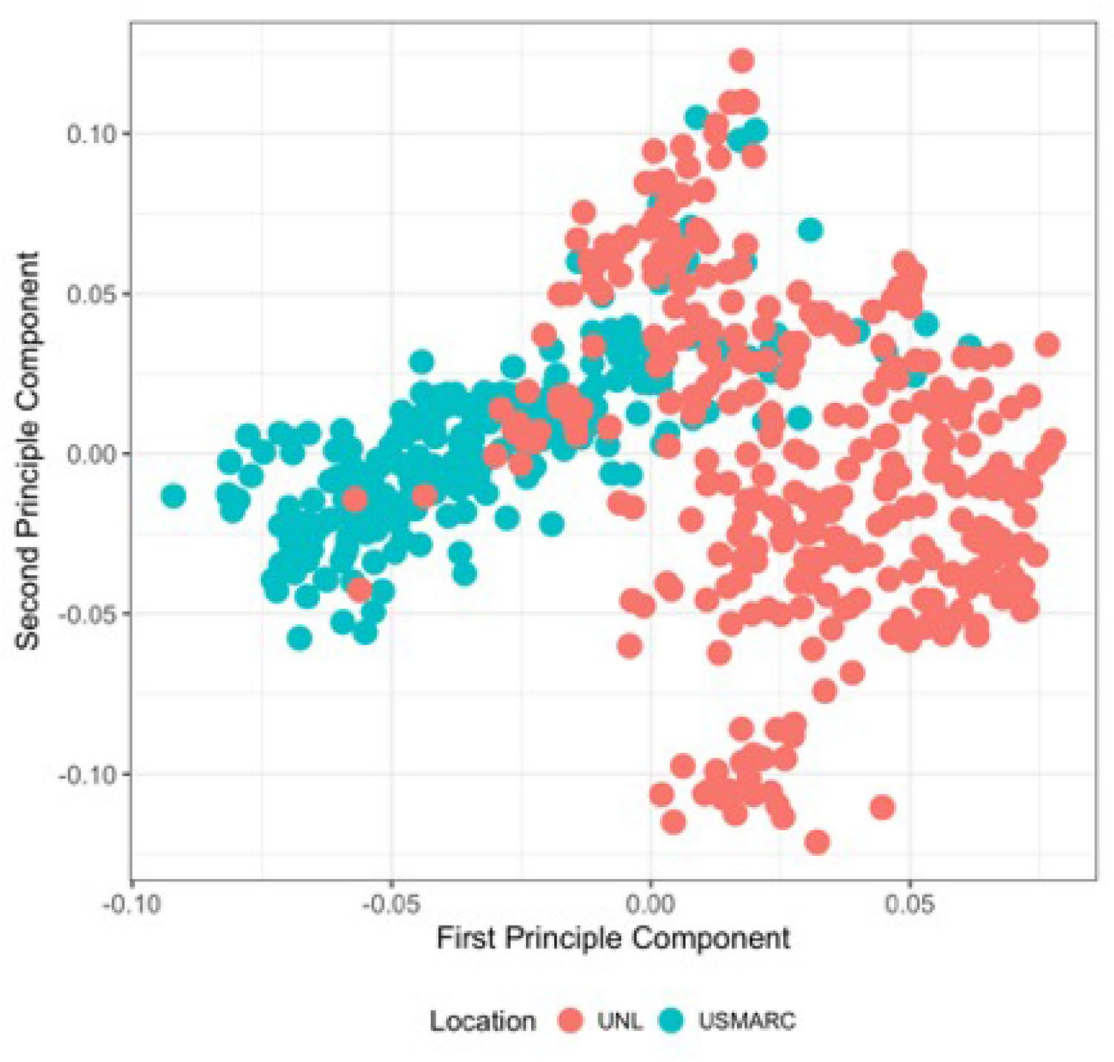
First and Second principle components of the genomic relationship matrix for USMARC and UNL animals demonstrating limited genomic variation in the two locations^1^. A genomic relationship matrix was constructed for all the animals sampled from USMARC and UNL using the SNP information. The principal component analysis (PCA) was run on genomic relationship matrix and first two principal components (PC1 and PC2) were plotted^1^. USMARC refers to animals that were fed at either the U.S. Meat Animal Research Center and UNL refers to animals fed at the University of Nebraska-Lincoln.

To determine the extent of and to correct for population structure, a principle component analysis (PCA) on a genomic relationship matrix (G) was utilized. The first two principle components annotated by location (UNL and USMARC) are illustrated in Fig. 2. The degree of population differentiation across the two feeding locations was minimal as the first principal component only described 3% of the variation in population structure. We utilized genome wide association analysis using SNP to assess the contribution of host genotype to the variation of bacterial taxa within the rumen. Normalized abundance of each OTU, family and phylum were used as traits to test for associations with 61,974 SNP.

**Figure 3:**
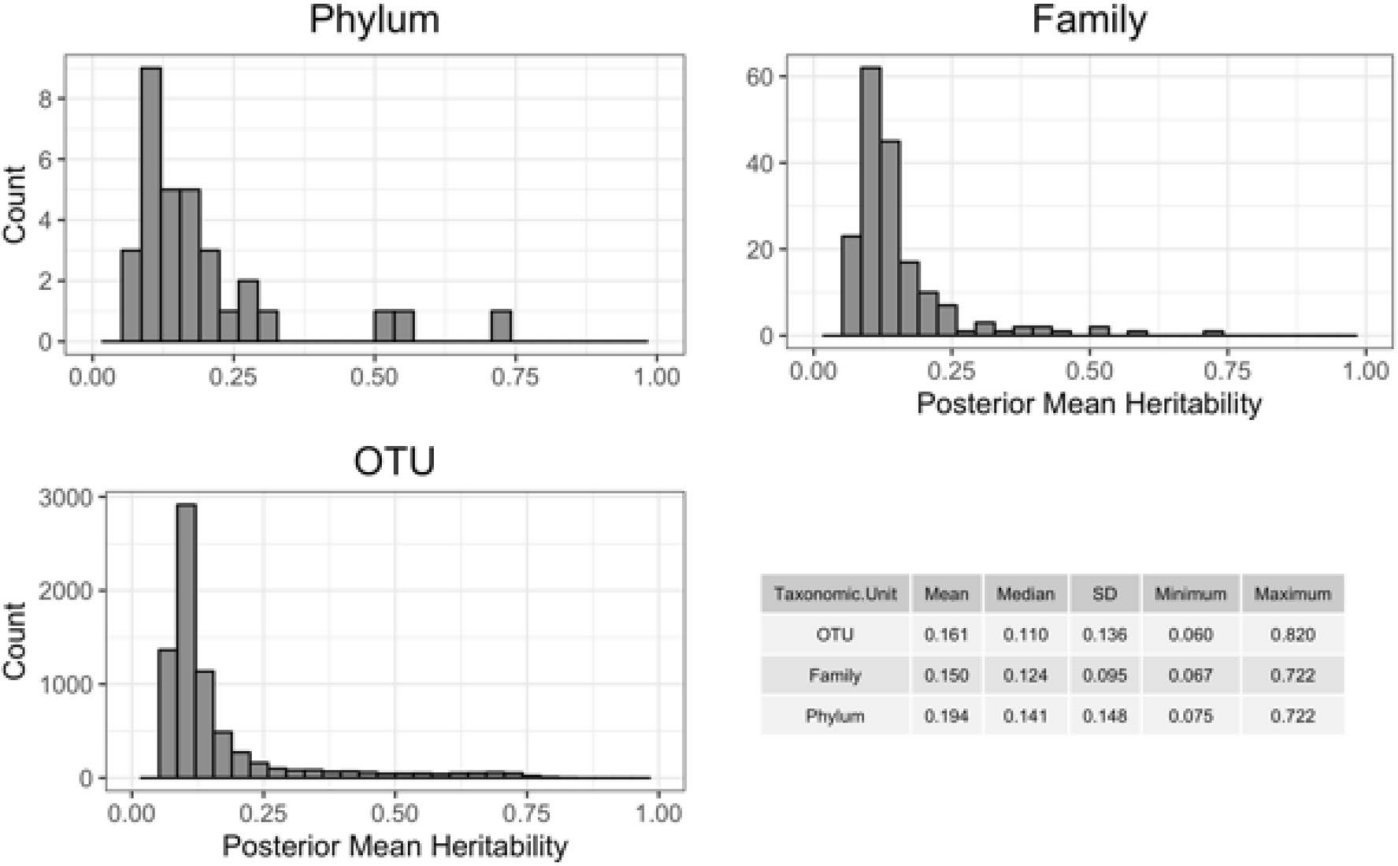
Posterior heritability estimates and summary statistics across taxa for phylum, family and operational taxonomic unit (OTU) categories. The relative abundances of Phyla, Families and OTUs were used to estimate the posterior heritability by using the GBLUP model (see “Methods”). The histograms represent the number of Phyla, Families and OTUs that fall into a specific range of posterior heritability. The table shows the mean, median, standard deviation and the minimum and maximum posterior heritability for different taxonomy levels.

The posterior mean heritability estimates determined using a Bayesian genomic best linear unbiased prediction (GBLUP) model across classes within OTU, family, and phylum level were 0.161, 0.150, and 0.194, respectively. Although posterior mean heritability estimates were low, some taxonomic groups displayed high heritability estimates. The maximum heritability estimates of OTU, family, and phylum level were, 0.820, 0.722, and 0.722, respectively (Fig. 3). Taxa belonging to phyla *Euryarchaeota, TM6* and *Proteobacteria* and families, *Methanobacteriaceae, Sphaerochaetaceae,* and *Succinivibrionaceae* had heritability estimates greater than 0.5. Additionally, 364 OTUs had heritability estimates higher than 0.5 (supplementary Tables S2, S3, and S4). The effective sample size and summary statistics of heritability estimates are shown in supplementary figure S1.

**Figure 4:**
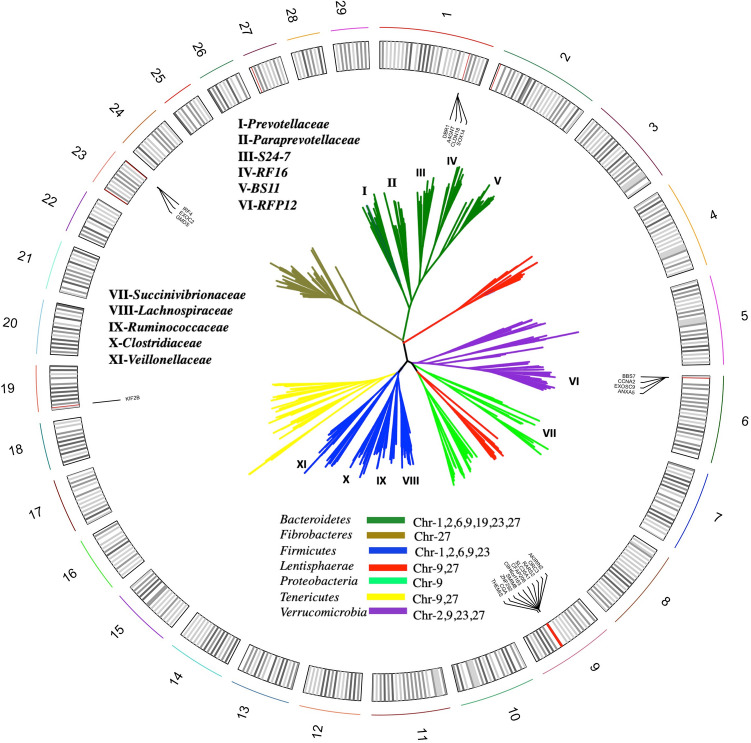
SNP mapping of the rumen gut microbiota. The circular diagram depicts the 29 bovine autosomes drawn to scale. Each black line represents 3 Mb region of the chromosome that includes the position of the SNPs used for Genome Wide Association Study. Red lines represent 1 Mb regions (Table 1) that have associations with different bacterial taxa in the rumen. The list of identified genes when annotated in the 1 Mb window are listed close to the region. A representative phylogenetic tree was generated from the rumen bacterial reads using the Interactive tree of life (iTOL). Major phyla are color-coded and associated chromosome(s) are listed with each phyla. The roman numerals represent families as follows; I—*Prevotellaceae*, II—*Paraprevotellaceae,* III—*S24-7,* IV—*RF16,* V—*BS11,* VI—*RFP12,* VII—*Succinivibrionaceae,* VIII—*Lachnospiraceae,* IX—*Ruminococcaceae,* X—*Clostridiaceae,* XI—*Veillonellaceae. *The complete list of annotated genes and their position on each chromosome is listed in supplementary Table S1.

For genome wide association analysis, the estimated additive genetic effects across animals were decomposed into marker effects across OTU, family, and phylum within each group. Chromosomal regions with the largest WGEBV for the selected OTU, family, and phylum are shown in Table 1. The identified SNP in the bovine genome and the phylogenetic relationship of the associated taxa are shown in Fig. 4. The 1-Mb chromosomal regions explained 0.32–3.24% (supplementary Table S5) of the phenotypic variation, where the largest variation was associated with OTU4 and family *Prevotellaceae*. Genomic associations were observed across diverse phylogenetic groups where associations were detected across 7 different phyla including the 4 major phyla that represent more than 90% of the rumen bacterial population (Fig. 4). The SNP associated with bacterial taxa were distributed across 7 autosomal chromosomes with chromosome 9 and 27 demonstrating associations at phylum, family and OTU level. Additionally, some taxa at OTU, family and phylum level were associated with 2 different SNP located on different chromosomes. As described previously^21^, this suggests the rumen microbiota is a heritable, polygenic trait.

**Table 1:** Chromosomal regions with the largest Window Genomic Estimated Breeding Value (WGEBV) for the selected OTUs, families, and phyla.

Class	Chromosome	Position (Mb; start–end)	Names
OTU	1	132.0–133.0	41, 57, 2,337
	2	2.0–3.0	21, 2,337, 43
	6	3.0–4.0	27, 30, 53
	9	63.0–64.0	4, 12, 53
	19	3.0–4.0	2,737, 3, 72
	23	0.0–1.0	12, 27, 2,737, 30, 4, 53, 72
	23	51.0–52.5	21, 64, 72
	27	3.0–4.0	19, 28, 3
Family	6	3.0–4.0	*BS11, Ruminococcaceae, Succinivibrionaceae*
	9	63.0–64.0	*Lachnospiraceae, RFP12, Succinivibrionaceae, Veillonellaceae, Clostridiaceae*
	23	0.0–1.0	*Paraprevotellaceae, Prevotellaceae, RFP12, S24-7, Ruminococcaceae*
	27	3.0–4.0	*RF16, S24-7, Fibrobacteraceae, Clostridiaceae*
Phylum	9	63.0–67.0	*Firmicutes, Lentisphaerae, Proteobacteria, Verrucomicrobia*
	27	3.0–4.0	*Fibrobacteres, Lentisphaerae, Tenericutes*

### Different taxonomic levels are under host genetic control

**Figure 5:**
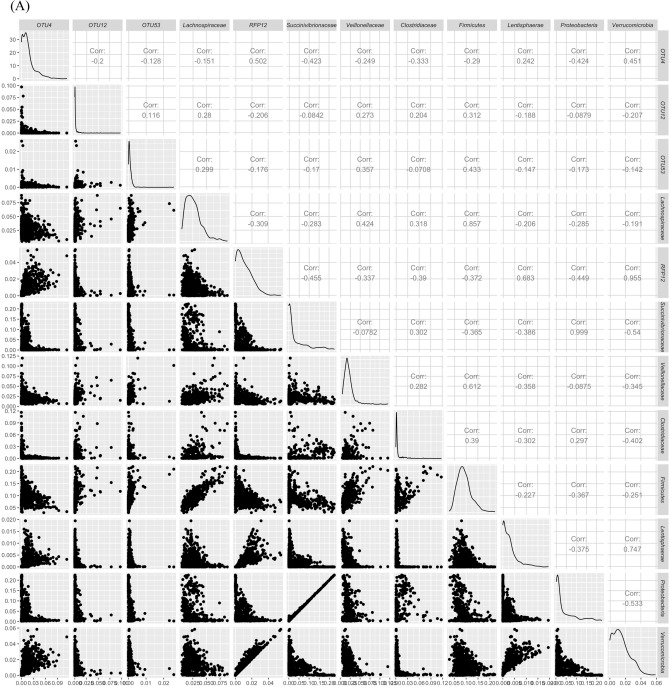

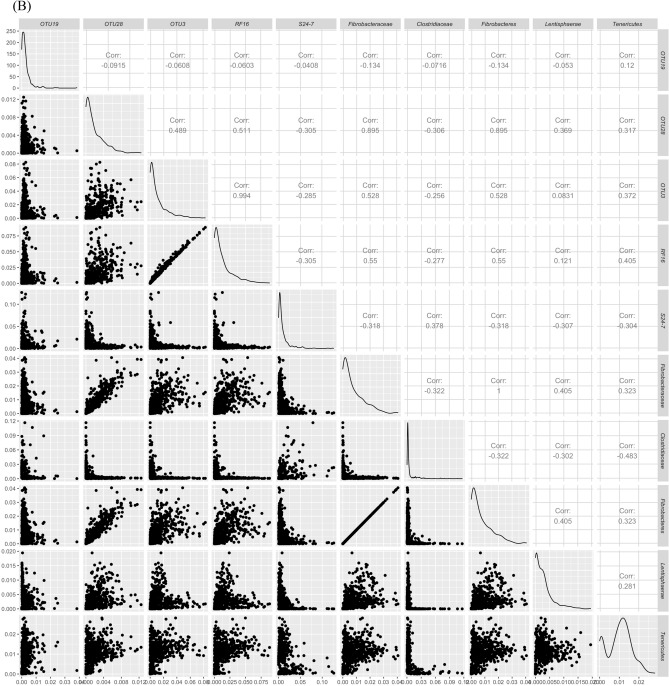
Correlations between OTU, Phyla and Family abundance associated with chromosome 9 (**A**) and chromosome 27 (**B**). A matrix was generated using log (1 + x) transformed relative abundances for OTUs, Phyla and Families associated with each chromosome and pairwise Pearson correlations were calculated and scatter plots and density plots were generated for each chromosome. The pairwise correlations among all the identified taxa on all chromosomes are shown in supplementary figure S3 and further taxonomic information of all identified OTUs can be found in Supplementary Table S1.

The majority of the associations were identified at the OTU level. However, phylum and family level associations were also detected on chromosomes 9 and 27 in addition to OTU associations. As such, to determine if higher taxonomic level associations detected are a result of higher abundance of a single OTU, we performed correlation analysis between taxa on chromosomes 9 and 27 (Fig. 5). Correlation analysis demonstrated that some phylum level associations are driven by one family belonging to that phylum (phylum *Fibrobacter *and family *Fibrobacteriaceae* r = 1.0; phylum *Proteobacteria* and family *Succinivibrionaceae* r = 0.999; phylum *Verrucomicrobia* and family *RFP12* r = 0.955). However, other phyla did not show such correlations with family level associations (*Firmicutes, Lentisphaerae, and Tenericutes*). A similar trend was seen between OTU level associations and family level associations where a few associations were highly correlated (OTU3 and family *RF16* r = 0.994, and OTU28 and family *Fibrobacteriaceae* r = 0.895) (supplementary Figure S2). However, a majority of the associations detected at OTU level were unique and did not have correlated families.

### Host adapted bacterial species in the rumen

Relatively large SNP associations were detected between genus *Methanobrevibacter*, *Succiniclasticum, Prevotella*, and *Fibrobacter*. Among the major genera associated with host genetics *Prevotella* was the most predominant genera. *Succiniclasticum* was significantly associated with both chromosome 1 and 2 similarly *Prevotella* was associated with chromosomes 2, 6, 9, 19, 23 and 27 (Table 1 and supplementary table S6). *Methanobrevibacter* was associated with chromosome 1 and *Fibrobacter* was associated with chromosome 27 (Table 1 and supplementary table S6). As described previously^21^, pleiotropic effects (having multiple effects from a single gene) were observed in many taxa. Including SNP that affect closely related taxa such as the associations observed on chromosome 9 for family *Veillonellaceae *and *Clostridiaceae*; and the association between *Prevotella* and *Paraprevotella* on chromosome 23. These observations suggest that genes in these 1 Mb regions can independently or in unity affect bacterial distribution and structure in the rumen. Additionally, the associated 1 Mb region on chromosome 27 is associated with the bacterial community composition of very diverse bacterial communities including phylum *Fibrobacteres, Lentisphaerae* and *Tenericutes*. As such, this genomic region can affect multiple diverse taxa within the rumen microbiome. Finally, we also detected genomic regions in multiple chromosomes that control the same bacterial genera. For example, OTU30 belonging to genus *Prevotella* was associated with 1 Mb regions in chromosomes 6, and 23. Additionally, OTU2337 belonging to genus *Succiniclasticum* was associated with 1 Mb regions in both chromosome 1 and 2. Similarly, OTU53 and OTU72 was associated with 1 Mb regions in chromosomes 6, 9, 23 and chromosomes 19, 23. OTU72 was associated with two different 1 Mb regions in chromosome 23.

## Discussion

The rumen microbiome has recently begun to be explored^1,25–27^. Similar to the mammalian intestinal microbiota, the cattle rumen microbiota is dominated by *Bacteriodetes, Firmicutes,* and *Proteobacteria* (based on type of diet)^14,15,25,26^. However, cattle rumen microbiota is distinct from intestinal microbiota of monogastric animals because it has a considerable proportion of members of the Phylum *Fibrobacter,* which are involved in fiber digestion, and because the microbial communities in the rumen utilize the diet before the host digestive system.

It is well established that diet is a major contributor of rumen microbial community composition^1,14,16,17^. However, in the current study cattle originated from two locations, whereby 2 of the 3 diets where only represented at one location. Furthermore, heifers only originated from one location and were fed a diet that differed from that fed to steers. This data structure, and the fact that samples were generated across multiple years, makes parsing these effects impossible. As such, to identify the collective effect of these factors on the rumen bacterial community, we performed PCoA analysis (supplementary figure S3) and PERMANOVA analysis using management type to reflect the collective effect diet, location, and sex. PERMANOVA analysis displayed a significant effect of management type on microbial community composition suggesting that collectively these factors affected the rumen microbial community composition. As the objective of this study was to identify association between host genetics and the rumen bacterial community composition, we fitted contemporary group, the concatenation of management type and year, in the model used for GWAS to adjust for the variation in the microbial community composition resulting from management type as described in methods. As such, the association observed in this study has been corrected for variations in the rumen bacterial community that can result from diet, sex, location and year.

Studies investigating the effect of host genetics on shaping the microbial community composition are limited. Recent studies in the bovine rumen have demonstrated that the rumen microbiome is influenced by host genetic factors^5,6,21^. As such, the concept of core taxa within the rumen microbiome being controlled by host genetics is intriguing as it lends to the potential to utilize associations between rumen microbiota and genetic markers for marker-assisted selection and management to improve feed efficiency, animal health and microbiome manipulation mediated by selecting for favorable microbial taxa within the rumen.

In this study, we demonstrate that the relative abundance of the most abundant taxa within the rumen microbiome is a “polygenic trait”^12^ under host genetic control. The pleiotropic effect of host genetics on the rumen microbiome demonstrates effects at multiple taxonomic levels. Data in this study demonstrates that the influence of host genetics in shaping the rumen microbiome is more effective at lower taxonomic units and in most cases the effect on the microbiome can be very specific. This is clearly demonstrated by the OTU level associations identified in this study. The association identified with the genus *Methanobrevibacter* on chromosome 1 is the first report of an association identified between an archaeal species in the rumen and the bovine host. We also identified family *Succinovibrionaceae*, which has been previously identified to be linked with methane emission^28^, associated with chromosome 9. A previous study by Wallace et al. identified *Succinovibrionaceae* to be heritable in dairy cows. Other studies have also reported host genetics and the microbiome to be associated with methane emission^29^ and this association further supports this notion. In the rumen, archaea play a critical role in recycling NADH back to NAD for glycolysis^2,30^, so that pyruvate in the rumen is spared for VFA production which becomes an energy source for the host^2,30^. In the absence of methanogens, recycling of NADH is performed by conversion of pyruvate to lactate or ethanol^2^, as such the pyruvate available for VFA production is wasted to recycle NADH making the rumen ecosystem less efficient. Therefore, the association observed between the host and the methanogen is directed toward increased energy to the host animal.

We observed associations on chromosome 1 and 2 for genus *Succiniclasticum.* Only one species named *Succiniclasticum ruminis* has been described in this genus^31^. *Succiniclasticum ruminis* is a common inhabitant in the rumen specialized in its ability to convert succinate to propionate as its sole mechanism of energy production. In the rumen, succinate is not accumulated as it is rapidly converted to propionate, and *Succiniclasticum ruminis* is considered as the major organism that is involved in this process. As, propionate is the only gluconeogenic volatile fatty acid in the rumen and also provides more ATP to the host than any other VFA produced in the rumen, it is not surprising that the host animal would prefer selective enrichment of microbes that increase energy supply to the host. As such the association between the host and the genus Succiniclasticum has important consequences towards host performance and well-being. The association observed for *Fibrobacter succinogenes* in chromosome 27 also provides evidence to support the notion that the host genome controls for microbes that help increase nutrient metabolism within the rumen. *Fibrobacter succinogenes* is a characterized as one of the key cellulolytic microbes in the rumen^32^, that help breakdown cellulose in the rumen. Genome sequence of *Fibrobacter succinogenes* S85^33^ has revealed that this organism contains 83 glycosyl hydrolases including 33 cellulases and 24 xylanases, 7 pectate lyases and 14 carbohydrate esterases^33,34^. As such *Fibrobacter succinogenes* is a key microbe in cellulose digestion in the rumen and the association with host genetics suggests that this organism is under genetic control to ensure efficient nutrient metabolism in the rumen and that the host animal’s energy requirements can be met from metabolites of microbial fermentation. Wallace et al.^21^ also reported the *Fibrobacter succinogenes* as one of the heritable bacteria in dairy cows.

We observed several loci on different chromosomes (2, 6, 9, 19, 23 and 27) to be associated with the genus *Prevotella. Prevotella* is a dominant genus found in the rumen and has been implicated in protein and energy metabolism in the rumen^1,14^. It is possible that each of the loci may be affecting the compositional changes of different species of *Prevotella* such as *P. ruminicola, P. bryanti, P. brevis,* and *P. albinensis* which are all been reported to be members of the rumen microbiome^35^. Although, we are unable to classify the OTUs identified at species level resolution, the fact that different OTUs belonging to genus *Prevotella* are associated with different loci suggests that different *Prevetella* species may be controlled by different loci. Recently, Li et al. reported 19 SNPs to be associated with 14 microbial taxa in the rumen^5^. This study also identified associations between *Paraprevotellaceae* and bovine chromosome 16, further supporting the notion of genus *Prevotella* may be controlled by many genetic loci. Similarly, family *Prevotellaceae* were reported as highly heritable among Nordic Red dairy cows^21^.

Describing factors that shape the rumen microbiome is important to improve animal health and performance and here we see a trend of the host selecting for bacterial species that help in nutrient metabolism in the rumen. To further investigate the associations between the taxa identified and the host, we performed “positional candidate gene analysis”. Positional gene candidate analysis helped identify genes located within the 1 Mb regions associated with rumen bacterial species (supplementary table S1 and S5). Many of the 1 Mb chromosomal regions identified did not contain annotated genes within the bovine genome. The only well annotated region in the bovine genome was in chromosome 9 (supplementary table S1). The 1 Mb regions between 63 and 67 Mb in chromosome 9 demonstrated association at OTU (63–64 Mb), family (63–64 Mb) and phylum (63–67 Mb) level controlling the abundance of both gram-positive and negative bacteria in the rumen. Positional candidate gene analysis in this region identified genes required for innate immunity, specifically AKIRIN2, a downstream effector of the Toll-like receptor (TLR), TNF and IL-1 beta signaling pathways that results in IL-6 production^36^. As such, it is possible that the association of rumen bacteria to this region of the host chromosome may result in cytokine secretion leading to modulation of host immunity. A similar association was observed on chromosome 9, 66–67 Mb region with the Thymocyte-Expressed Molecule Involved In Selection (THEMIS) gene. This gene has been described to play a critical role in thymocyte development and maturation of T-cells^37^ further implicating the interaction between the host genetics and the rumen microbiome in immune modulation.

Additionally, the rumen epithelium is characterized as a stratified squamous epithelium, and has been described as an organ involved in selective absorption of nutrients in the form of volatile fatty acids (VFAs) from rumen bacterial fermentation^38^. Therefore, active and secondary active transport systems mediate nutrient absorption^38,39^. Previous studies have demonstrated Claudin family genes to play a role in the formation of the permeability barrier and to help with tight junction formation in the rumen^38^. In our GWAS, we identified a significant association with claudin-18 (CLDN18) in chromosome 1 in the 132–133 Mb region. This region was associated with the specialized propionate producer *Succiniclasticum ruminis* that utilizes conversion of succinate to propionate as its sole energy producing reaction^31^. As such, it is tempting to speculate that this association between claudin-18 and *Succiniclasticum ruminis* is involved in selective VFA absorption to the host. In addition to the described associations above, associations with GDP-Mannose 4,6-Dehydratase (GMDS) in chromosome 23, Alpha-1,4-N-Acetylglucosaminyltransferase (A4GNT) in chromosome 1, and ANXA5 involved in endocytotic and exocytotic pathways in chromosome 6 all suggests implication of the host in modulating the rumen microbes to increase energy absorption. A majority of the SNPs identified by Li et al. were present in the non-coding region and thus they were unable to identify how the associated taxa may influence host metabolism or health. The few genes identified in that study suggested, the associated regions to increase nutrient absorption to the host. Results presented in this study is consistent with this notion and the associations observed between the host and the microbial species further suggests that the associations between the microbiota and host genotype is directed toward selective absorption of volatile fatty acids from the rumen to increase energy availability to the host animal.

In the ruminant animal, 50–70% of the animal’s protein needs and up to 70% of the energy needs^7^ are met through the metabolism of rumen microbes. The rumen microbiota can be viewed as an environmental factor that impacts animal health, nutrition and performance. Studies have demonstrated the rumen bacterial species composition to influence feed efficiency, Average Daily Gain (ADG) and intake^40^. As such demonstrating that heritable trait in the host genome can impact rumen bacterial species composition provides new opportunities to using genome selection to improve animal health and productivity. Future work evaluating the SNP identified herein and their relative effects in other populations as well as the recruitment of associated bacterial taxa in subsequent generations when parents are selected based on genotypes at these loci would be interesting. Such investigations may lead to the possibility of selection for microbiome manipulation. Additionally, similar associations may exist between the host genotype and the fungal, protozoal and viral populations within the rumen. Future studies focused on other members of the rumen microbiome may shed light into the role of host genetics shaping these microbial populations.

## Materials and methods

### Experimental design and animals

All animal procedures implemented in this study were approved by the University of Nebraska—Lincoln and U.S. Meat Animal Research Center (USMARC) Animal Care and Use Committee. All experiments were performed using relevant guidelines and regulations described by the Animal Care and Use Committee. The data presented in this study were collected between 2009 and 2015 from different cohorts of animals which included a cohort of heifers (*n* = 127) and a cohort of steers (*n* = 131) from USMARC (n = 258) and a cohort of steers (n = 328) from University of Nebraska, Lincoln (UNL) research feedlot. Animals within USMARC and UNL were fed in 3 (USMARC1-3) and 5 (UNL1-5) cohort groups, respectively. The number of animals within each cohort ranged from 51 to 127 for animals fed at USMARC and 51 to 111 for animals fed at UNL. The animals from USMARC were part of the USMARC Germplasm Evaluation project (GPE)^41^. The animals from UNL were purchased from Nebraska and surrounding states and were a cross bred population of unknown breed makeup.

The Heifers at USMARC were fed a growing diet at time of sample collection that included 70% corn silage and 30% alfalfa hay on dry matter basis^40^. The USMARC steers were fed a finishing diet composed of 57.6% dry-rolled corn, 30% wet distillers grains with solubles, 8% alfalfa hay, and 4.4% vitamin and mineral supplement on a dry matter basis^40^. All UNL animals were fed a common basal diet (UNL-Diet) containing 50:50 blend of Alfalfa and Sweet Bran®. After adaptation to each diet for at least 21 days, rumen samples were collected via esophageal tubing for bacterial community analysis. The diets were formulated to meet or exceed NRC recommendations for growth and vitamin and mineral supplementation of growing and finishing beef cattle.

### Sample collection for rumen microbiota composition analysis

Rumen samples were collected after adaptation to each diet via esophageal tubing as described previously^42^. Briefly, the animal was restrained in the chute, and a stomach tube was inserted through a speculum and passed through the esophagus until it reached the rumen. A vacuum pump was attached to the free end of the tube and the sample was withdrawn from the rumen. The samples collected contained both rumen fluid and feed particles and were a representative sample of the rumen. Samples collected via esophageal tubing have been shown to represent the rumen to contain a similar microbial community composition to a sample collected via a rumen fistula^42^. The samples collected were snap frozen in liquid nitrogen and were stored in − 80 °C until used for DNA extraction and microbial community analysis.

### Microbial DNA extraction

DNA was extracted from the rumen samples using the PowerMag Soil DNA isolation kit (MoBio Laboratories, Carlsbad, CA, USA) according to the manufacturer’s protocol with a few modifications as described by Paz et al.^40^. The modified protocol also included adding RNase A to the lysis solution to ensure removal of RNA during DNA extraction. The isolated DNA was stored at − 20 °C until used for bacterial community analysis.

The V4 region of the 16S rRNA gene was amplified and sequenced on the MiSeq platform as described previously^40,43^. Briefly, barcoded universal primers specific for the V4 region of eubacteria were amplified using 25 μL PCR amplification reactions^43^. Each 25 μL PCR reaction contained 0.75 Units Terra PCR Direct Polymerase Mix, 1X Terra PCR Direct Buffer, 10 μM indexed fusion primers, and 5–20 ng of DNA. The cycling conditions contained 98 °C for 2 min, followed by 25 cycles of 98 °C for 10 s, 55 °C for 30 s, and 68 °C for 30 s; and a final elongation step of 68 °C for 4 min^40^. Following amplification, PCR products were normalized using the SequalPrep Normalization Plate Kit (Invitrogen, Carlsbad, CA, USA) according to the manufacturers protocol. The normalized libraries were pooled and were further purified using the MinElute PCR Purification Kit (Qiagen, Valencia, CA, USA) as described by the manufacturer. The resulting pooled sample was subjected to size selection and purification using the Pippin Prep (Sage Science, Inc., Beverly, MA, USA) automated size selection instrument. The resulting sequence ready libraries were further analyzed using the Agilent BioAnalyzer 2100 (Agilent Technologies, Santa Clara, CA, USA) and were subjected to 250 bp paired end sequencing using the Illumina Miseq System (Illumina, San Diego, CA, USA) using the V2 500 cycle sequencing kit as described by the manufacturer. Bridge amplification and reversible dye-terminator -based sequencing on the MiSeq was performed as described by the manufacturer. Raw sequences have been deposited at the NCBI Sequence Read Archive (SRA) under the accession no. SRP100776 and PRJNA55259.

### Data processing pipelines for microbial community analysis

Raw reads generated from Illumina MiSeq sequencing were processed using the quality filter and analysis pipeline described by Paz et al.^40^. Following preliminary quality filtering and read processing, the resulting reads were used for microbial community analysis as described below. Complete information of the bioinformatics pipeline describing data analysis is available at https://github.com/FernandoLab. Briefly, forward and reverse reads were assembled to generate contigs of the V4 region and further quality filtering was performed on subsequent contigs to remove sequences with ambiguous bases, incorrect contig length, or incorrect assembly using MOTHUR v.1.38.1^23^. Following secondary quality filtering, subsequent reads were clustered into operational taxonomic units (OTUs) using the UPARSE pipeline (USEARCH v7.0.1090)^44^ after dereplication, discarding singletons, clustering sequences into OTUs at 97% similarity, and removing chimeric sequences using UCHIME^45^ as described previously^40^. Representative OTU sequences from each OTU were aligned against the SILVA reference alignment v123 to ensure the OTU reads came from the V4 region of the 16S rDNA gene. The resulting representative OTU sequences that fail to map to the V4 region were discarded. Taxonomy was assigned to each OTU using QIIME v.1.9.1^46^ as described previously^23^. The Greengenes database (gg_13_8_otus)^24^ were used as the reference database for taxonomic assignment. OTUs classified as Cyanobacteria were filtered from the data set as cyanobacterial sequences may arise from 16S remnants present in the plant chloroplasts^47^. However, recent studies have reported Cyanobacterial orders such as YS2, SM1D11, and mle1-12 to be a new class of Cyanobacteria to be present in gut, soil and plants^48–50^. However, these orders were not identified within the cyanobacterial sequences that were removed. A rarefied (7,000 reads) OTU table was used to generate a Bray Curtis dissimilarity matrix that was used for Principal Coordinate Analysis (PCoA) within QIIME v.1.9.1 pipeline. The R package ggplot2^51^ was used to generate the PCoA plot in R by using first two components of the Bray Curtis coordinates. The Bray Curtis dissimilarity matrix was used to perform the multivariate analysis of variance (PERMANOVA)^52^ within R package vegan^53^. The complete information of the bioinformatics pipeline describing data analysis is available at https://github.com/FernandoLab.

### Genotyping the resource population

All animals (n = 586) were genotyped with either a 770 K, 150 K, or 80 K SNP assay. Blood samples were collected onto blood collection cards and the collection cards were sent to Geneseek (Lincoln, NE) for subsequent DNA extraction and genotyping. The SNP in common across all panels were utilized in the analysis and included 61,974 SNP. Genotypes that were missing were replaced with the mean genotype at that locus across all genotyped individuals.

### Identifying “positional candidate genes”

The 1 Mb windows that account for the greatest proportion of the genetic variance were used to identify candidate genes using the “positional candidate gene approach” using the Bos taurus build UMD_3.1 assembly^54^ and UCSC genome (ars-ucd1.2/bos taurus) browser. Due to the limited understanding of the Bos taurus genome compared to Homo sapiens, human orthologs of beef cattle “positional candidate genes” was identified using the BioMart data mining tool (68) and the Ensembl Genes (release 69). Human gene orthologs to bovine “positional candidate genes” was utilized to identify gene ontology terms, and pathways using the UCSC genome (ars-ucd1.2/bos taurus) browser and NCBI gene functions.

### Statistical analysis

An OTU table generated for the 586 animals was used for subsequent analysis. Rarefaction analysis was performed using QIIME v.1.9.1^46^ as described previously^40^. Prior to the analysis, operational taxonomic unit (OTU), family and phylum read counts were transformed into relative abundance.

The heritability of an OTU, family, or phylum class was estimated using a Bayesian genomic best linear unbiased prediction (GBLUP) model utilizing the BGLR package in R^55^. Within each OTU, family, and phylum the following model was fitted:

$$\mathrm{y}= \mathbf{X}\mathrm{b}+\mathbf{Z}\mathrm{u}+ \text{e},$$

where y is the proportional abundance, b is a vector of fixed effects, u is a vector of random additive genetic effects, e is a vector of random residuals and X and Z are incidence matrices relating observations to the fixed and random additive genetic effects, respectively. To determine the extent of and to correct for population structure, a principle component analysis (PCA) on a genomic relationship matrix (G) was utilized. The G matrix was constructed as:

$$\mathbf{G}=\frac{{\varvec{M}}{\varvec{M}}\mathrm{^{\prime}}}{2\sum {p}_{j}(1-{p}_{j})},$$

where M is a genotype incidence matrix that has been centered based on allele frequencies (VanRaden, 2008) and p is the allele frequency of the second allele at the jth SNP across all loci.

The fixed effects included the intercept, the first two PC, and contemporary group (concatenation of management type and year).

The random additive genetic effect was assumed ~ N(0,${\mathbf{G}\sigma }_{u}^{2})$, where $\mathbf{G}$ is a genomic relationship as outlined previously. A chain length of 102,000 iterations was run with the first 2000 discarded as burn-in and a thinning rate of 10 was utilized. The default priors were utilized within BGLR, which included a flat and bounded prior for the fixed effects and a scaled inverse Chi-squared distribution for the additive genetic and residual variances. Across both random effects the degrees of freedom were set at their default value of 5 and the scale factors were set based on the rules described by de los Campos and Pérez-Rodríguez^56^ with the assumption that the model explains 20% of the phenotypic variance. The posterior mean $\pm \mathrm{ PSD}$ heritiability estimates for a given OTU, family, or phylum was calculated as the mean of $\frac{{\sigma }_{u}^{2}}{{\sigma }_{u}^{2}+{\sigma }_{e}^{2}}$ across all samples that remained after thinning. The number of effective samples across models was estimated using the CODA package in R^57^.

### Genome-wide association study

Across OTU, family, or phylum class the estimated additive genetic effects across animals were decomposed into marker effects for a subset of groups within each class^58^. The classes investigated are outlined in Table S1. The marker effects (a) were estimated by backsolving using G and the incidence matrix (i.e., Z) as outlined below:

$${\hat{\rm{a}} }=\frac{{{\varvec{M}}}^{\mathrm{^{\prime}}}{{\varvec{G}}}^{-1}\widehat{u}}{2\sum {p}_{j}(1-{p}_{j})},$$

where M is the genotype incidence matrix as outlined previously, ${{\varvec{G}}}^{-1}$ is the inverse of G and $\widehat{u}$ is the estimated additive genetic value of an individual which was derived from the Bayesian GBLUP model. After estimating marker effect solutions, the variance of 1 Mb non-overlapping window genomic estimated breeding values (WGEBV) was computed for each window. Within a window the WGEBV for each individual was estimated by multiplying the estimated SNP effects with their respective genotypes and summing across all SNP within the window. The WGEBV variance of a 1-Mb window was calculated as the variance in WGEBV across individuals. All OTUs (n = 17) with at least 40,000 reads and present in at least 95% of the animals, all families (n = 12) that represented greater than 1.8% total reads and were the top families (12 families were selected as families ranked 11th and 12th had very similar read percentages to the family ranked 10th) and top 10 Phyla (n = 10) with the largest variance in WGEBV were investigated further. The relative abundances of the identified OTUs, families and phyla were transformed with natural log (log(1 + x)) and then pairwise Pearson correlation analyses were performed. The pairwise correlation among OTUs, families and Phyla associated with chromosome 9 and 27 were plotted using R package GGally^59^ while correlation heatmap among all the identified OTUs, families and phyla was generated by using R package ggplot2^51^.

## Supplementary information

Supplementary Information 1.

Supplementary Information 2.

Supplementary Information 3.

Supplementary Information 4.

## Data Availability

Raw sequences generated in this study have been deposited at the NCBI Sequence Read Archive (SRA) under the accession no. SRP100776 and PRJNA55259. The complete information of the bioinformatics pipeline describing data analysis is available at https://github.com/FernandoLab.
