# Supplementary material for: Influence of host genetics in shaping the rumen bacterial community in beef cattle
Source: Sci Rep. 2020 Sep 15;10:15101. doi: 10.1038/s41598-020-72011-9 (PMC7493918; doi:10.1038/s41598-020-72011-9)
Supplement: Supplementary file 1 — Supplementary Information 1. [file 41598_2020_72011_MOESM1_ESM.docx]

**Supplementary Information:**

Title: Influence of host genetics in shaping the rumen bacterial community in beef cattle

Running title: Rumen microbiome is influenced by host genetics

Waseem Abbas**^+^**^1^, Jeremy T. Howard**^+^**^1^, Henry A. Paz^1,2^, Kristin E. Hales^3^, James E. Wells^3^, Larry A. Kuehn^3^, Galen E. Erickson^1^, Matthew L. Spangler^1^**^*^**  , Samodha C. Fernando^1^**^*^**

**^+^ These authors contributed equally to this work**

**^1^***Department of Animal Science, University of Nebraska-Lincoln, Lincoln, NE, USA,*

**^2^**Department of Animal and Dairy Science, Mississippi State University, MS, USA,

**^3^**Meat Animal Research Center, Clay Center, NE 68933,

**^*^** Correspondence:

M.L. Spangler, Department of Animal Science, University of Nebraska-Lincoln, Lincoln, NE, USA, Phone: 402-472-6489, Fax: 402-472-6362, E-mail: [mspangler2@unl.edu](mailto:mspangler2@unl.edu)

S. C Fernando, Department of Animal Science, University of Nebraska-Lincoln, Lincoln, NE, USA, Phone: 402-472-0518, Fax: 402-472-6362, E-mail: samodha@unl.edu

Mention of trade names or commercial products in this article is solely for the purpose of providing specific information and does not imply recommendation or endorsement by the USDA. USDA is an equal opportunity provider and employer.

Samodha C. Fernando, author of this publication has disclosed a significant financial interest in NuGUT LLC. In accordance with its Conflict of Interest policy, the University of Nebraska-Lincoln’s Conflict of Interest in Research Committee has determined that this must be disclosed. The rest of the authors have nothing to disclose.

**
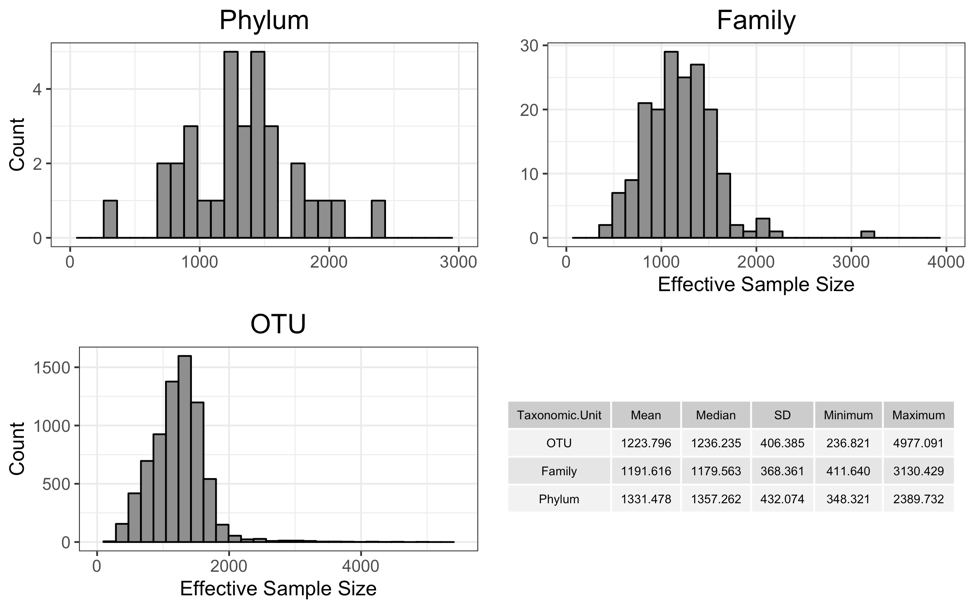
**

**Figure S1: Effective Sample size and summary statistics across taxa for phylum, family and operational taxonomic unit (OTU) categories.**


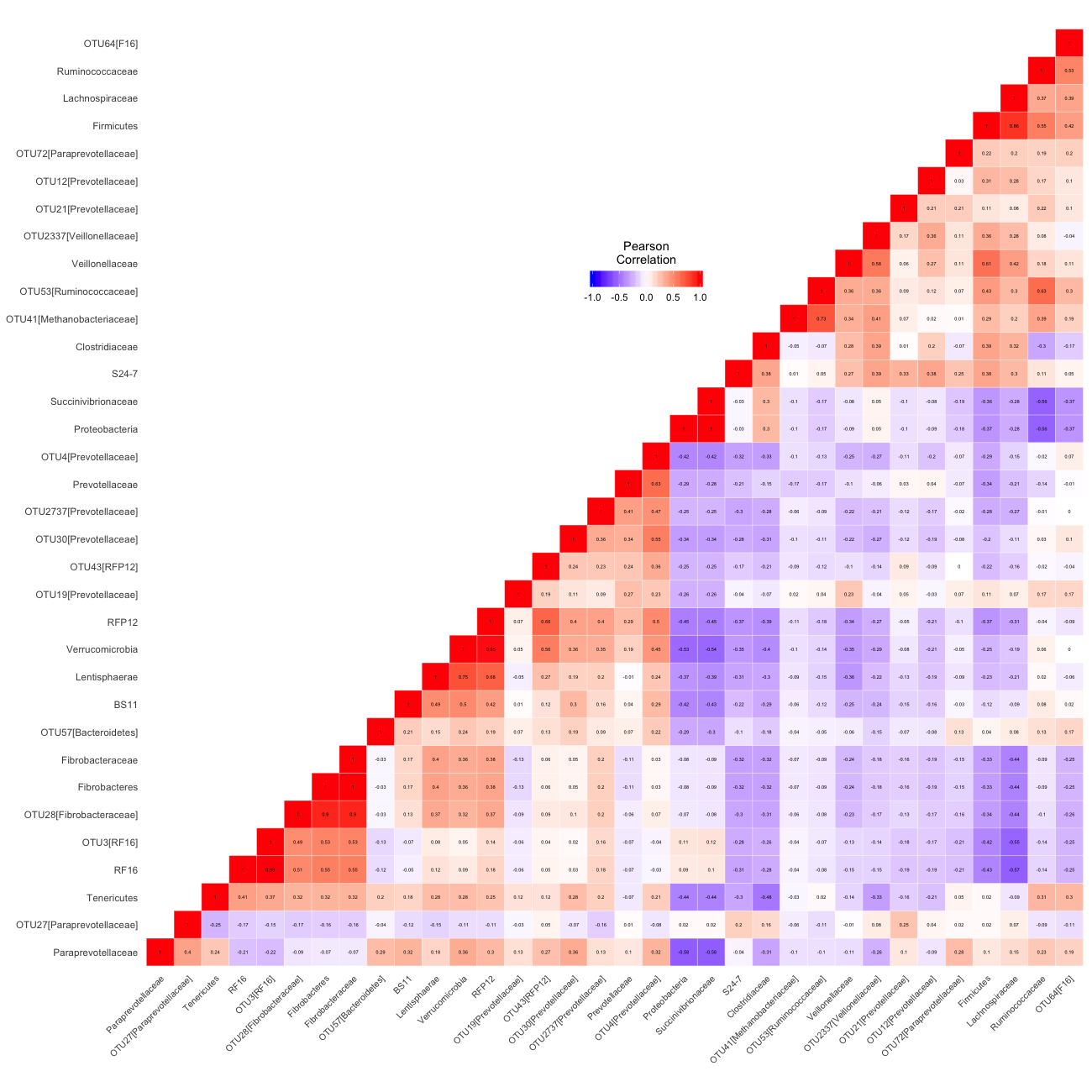


**Figure S2: Pairwise correlation between all the identified OTUs, Phyla and Families.**

A matrix was generated using log (1+x) transformed relative abundances for OTUs, Phyla and Families associated with each chromosome and pairwise Pearson correlations were calculated. The OTUs are listed and classified with Family they belong to. Detailed taxonomy for each OTU can be found in Supplementary Table S2.

**Figure S3: The PCoA plot based on bray Curtis dissimilarity matrix demonstrating the collective effect of Management type (diet, location, and sex).** All the samples were rarefied to 7000 reads. Blue – USMARC steers on a finishing diet, Green – USMARC Heifers on a growing diet and Red – UNL steers on a basal diet. Permutational analysis of variance PERMANOVA was performed using “management type” to reflect the collective effect of all above factors. PERMANOVA analysis identified significant differences between “management types” (*P<0.001*) in the bacterial community.

**Table S1: The known annotated genes and associated taxa**

| **Chromosome** | **chromStart** | **chromEnd** | **Gene** | **Taxonomy** |
| --- | --- | --- | --- | --- |
| chr1 | 132002980 | 132014615 | DBR1 | OTU41, OTU57, OTU2337 |
| chr1 | 132063621 | 132068263 | A4GNT |  |
| chr1 | 132174469 | 132191685 | CLDN18 |  |
| chr1 | 132449821 | 132450544 | SOX14 |  |
| chr6 | 3372938 | 3411895 | BBS7 | OTU21, OTU30, OTU53 |
| chr6 | 3413085 | 3419196 | CCNA2 |  |
| chr6 | 3419544 | 3432299 | EXOSC9 |  |
| chr6 | 3542634 | 3575330 | ANXA5 |  |
| chr9 | 63127338 | 63150182 | AKIRIN2 | OTU4, OTU12, OTU53, Lachnospiraceae, RFP12, Succinivibrionaceae, Veillonellaceae, Firmicutes, Lentisphaerae, Proteobacteria, Verrucomicrobia |
| chr9 | 63155999 | 63225311 | ORC3 |  |
| chr9 | 63225446 | 63299358 | RARS2 |  |
| chr9 | 63302745 | 63334395 | SLC35A1 |  |
| chr9 | 63346329 | 63382321 | CFAP206 |  |
| chr9 | 63430800 | 63458705 | C9H6orf163 |  |
| chr9 | 63462320 | 63474252 | SMIM8 |  |
| chr9 | 63520826 | 63622497 | ZNF292 |  |
| chr9 | 63677990 | 63694859 | CGA |  |
| chr9 | 63762921 | 63764019 | HTR1E |  |
| chr9 | 64765557 | 64849621 | SNX14 |  |
| chr9 | 64859549 | 64871772 | NT5E |  |
| chr9 | 64879263 | 64929945 | NT5E |  |
| chr9 | 65579317 | 65608088 | TBX18 |  |
| chr9 | 66115461 | 66211054 | CEP162 |  |
| chr9 | 66223875 | 66287509 | MRAP2 |  |
| chr9 | 66377195 | 66503206 | CYB5R4 |  |
| chr9 | 66683788 | 66885925 | THEMIS |  |
| chr9 | 66968685 | 67594209 | PTPRK |  |
| chr19 | 3876961 | 3879199 | KIF2B | OTU2737, OTU3, OTU72 |
| chr23 | 51115435 | 51453575 | GMDS | OTU21, OTU64, OTU72, Paraprevotellaceae, Prevotellaceae, RFP12, S24-7, Ruminococcaceae |
| chr23 | 51917494 | 52015703 | EXOC2 |  |
| chr23 | 52050984 | 52063706 | IRF4 |  |
|  |  |  |  |  |

**Table S5: Taxa location and variation explained for each genome wide association.**

| Taxonomy | Chromosome | Position  (Mb; Start –End) | Percent Variance Explained |
| --- | --- | --- | --- |
| OTU41 | Chr1 | 132.0-133.0 | 0.44 |
| OTU57 | Chr1 | 132.0-133.0 | 0.38 |
| OTU2337 | Chr1 | 132.0-133.0 | 0.40 |
| OTU21 | Chr2 | 2.0-3.0 | 0.55 |
| OTU2337 | Chr2 | 2.0-3.0 | 0.33 |
| OTU43 | Chr2 | 2.0-3.0 | 0.37 |
| OTU27 | Chr6 | 3.0-4.0 | 0.32 |
| OTU30 | Chr6 | 3.0-4.0 | 0.42 |
| OTU53 | Chr6 | 3.0-4.0 | 0.55 |
| OTU4 | Chr9 | 63.0 – 64.0 | 1.17 |
| OTU12 | Chr9 | 63.0 – 64.0 | 0.62 |
| OTU53 | Chr9 | 63.0 – 64.0 | 0.36 |
| OTU2737 | Chr19 | 3.0-4.0 | 0.57 |
| OTU3 | Chr19 | 3.0-4.0 | 0.44 |
| OTU72 | Chr19 | 3.0-4.0 | 0.37 |
| OTU12 | Chr23 | 0.0 – 1.0 | 0.73 |
| OTU 27 | Chr23 | 0.0 – 1.0 | 0.58 |
| OTU 2737 | Chr23 | 0.0 – 1.0 | 0.72 |
| OTU 30 | Chr23 | 0.0 – 1.0 | 0.40 |
| OTU 4 | Chr23 | 0.0 – 1.0 | 3.24 |
| OTU 53 | Chr23 | 0.0 – 1.0 | 1.66 |
| OTU 72 | Chr23 | 0.0 – 1.0 | 2.2 |
| OTU21 | Chr23 | 51.0– 52.5 | 1.09 |
| OTU64 | Chr23 | 51.0– 52.5 | 0.39 |
| OTU72 | Chr23 | 51.0– 52.5 | 0.42 |
| OTU19 | Chr27 | 3.0-4.0 | 0.85 |
| OTU28 | Chr27 | 3.0-4.0 | 0.48 |
| OTU3 | Chr27 | 3.0-4.0 | 1.15 |
| BS11 | Chr6 | 3.0-4.0 | 0.35 |
| Ruminococcaceae | Chr6 | 3.0-4.0 | 0.47 |
| Succinivibrionaceae | Chr6 | 3.0-4.0 | 0.44 |
| Lachnospiraceae | Chr9 | 63.0-64.0 | 1 |
| RFP12 | Chr9 | 63.0-64.0 | 0.63 |
| Succinivibrionaceae | Chr9 | 63.0-64.0 | 0.65 |
| Veillonellaceae | Chr9 | 63.0-64.0 | 1.03 |
| Clostridiaceae | Chr9 | 63.0-64.0 | 0.40 |
| Paraprevotellaceae, | Chr23 | 0.0 – 1.0 | 0.76 |
| Prevotellaceae | Chr23 | 0.0 – 1.0 | 2.06 |
| RFP12 | Chr23 | 0.0 – 1.0 | 0.37 |
| S24-7 | Chr23 | 0.0 – 1.0 | 1.71 |
| Ruminococcaceae | Chr23 | 0.0 – 1.0 | 0.43 |
| RF16 | Chr27 | 3.0 – 4.0 | 1.00 |
| S24-7 | Chr27 | 3.0 – 4.0 | 0.35 |
| Fibrobacteraceae | Chr27 | 3.0 – 4.0 | 0.70 |
| Clostridiaceae | Chr27 | 3.0 – 4.0 | 0.42 |
| Firmicutes | Chr9 | 63.0-67.0 | 1.13 |
| Lentisphaerae, | Chr9 | 63.0-67.0 | 0.60 |
| Proteobacteria, | Chr9 | 63.0-67.0 | 0.66 |
| Verrucomicrobia | Chr9 | 63.0-67.0 | 0.49 |
| Fibrobacteres | Chr27 | 3.0-4.0 | 0.71 |
| Lentisphaerae | Chr27 | 3.0-4.0 | 0.74 |
| Tenericutes | Chr27 | 3.0-4.0 | 0.37 |

**Table S6: Classification of OTUs Identified**

| **Chromosome** | **OTU** | **OTU Taxonomy** |
| --- | --- | --- |
| Chromosome 1 [132.0-133.0] | OTU41 | OTU41_Genus _Methanobrevibacter |
|  | OTU57 | OTU57_ Order_Bacteroidales |
|  | OTU2337 | OTU2337_Genus_ Succiniclasticum |
| Chromosome 2 [2.0-3.0] | OTU21 | OTU21_ Genus_Prevotella |
|  | OTU2337 | OTU2337_Genus_Succiniclasticum |
|  | OTU43 | OTU43_Family_RFP12 |
| Chromosome 6 [3.0-4.0] | OTU27 | OTU27_ Genus_YRC22 |
|  | OTU30 | OTU30_ Genus_Prevotella |
|  | OTU53 | OTU_53_ Family_Ruminococcaceae |
| Chromosome 9 [63.0 – 64.0] | OTU4 | OTU4_Genus_Prevotella |
|  | OTU12 | OTU12_Genus_Prevotella |
|  | OTU53 | OTU53_Family_Ruminococcaceae |
| Chromosome 19 [3.0-4.0] | OTU2737 | OTU2737_Genus_Prevotella |
|  | OTU3 | OTU3_Family_RF16 |
|  | OTU72 | OTU72_Genus_CF231 |
| Chromosome 23 [0.0 – 1.0] | OTU12 | OTU12_Genus_Prevotella |
|  | OTU27 | OTU27_Genus_YRC22 |
|  | OTU2737 | OTU2737_Genus_Prevotella |
|  | OTU30 | OTU30_Genus_Prevotella |
|  | OTU4 | OTU4_Genus_Prevotella |
|  | OTU_53 | OTU53_Family_Ruminococcaceae |
|  | OTU72 | OTU72_ Genus_CF231 |
| Chromosome 23 [51.0– 52.5] | OTU21 | OTU21_Genus_Prevotella |
|  | OTU64 | OTU64_Family_F16 |
|  | OTU72 | OTU72_Genus_CF231 |
| Chromosome27 [3.0-4.0] | OTU19 | OTU19_Genus_Prevotella |
|  | OTU28 | OTU28_Species_succinogenes |
|  | OTU3 | OTU3_Family_RF16 |
